# Deep-learned 3D black-blood imaging using automatic labelling technique and 3D convolutional neural networks for detecting metastatic brain tumors

**DOI:** 10.1038/s41598-018-27742-1

**Published:** 2018-06-21

**Authors:** Yohan Jun, Taejoon Eo, Taeseong Kim, Hyungseob Shin, Dosik Hwang, So Hi Bae, Yae Won Park, Ho-Joon Lee, Byoung Wook Choi, Sung Soo Ahn

**Affiliations:** 1 School of Electrical and Electronic Engineering, Yonsei University, Seoul, Korea; 2 Department of Radiology, Severance Hospital, Research Institute of Radiological Science and Center for Clinical Image Data Science, Yonsei University College of Medicine, Seoul, Korea; 3 Department of Radiology, Ewha Womans University College of Medicine, Seoul, Korea

## Abstract

Black-blood (BB) imaging is used to complement contrast-enhanced 3D gradient-echo (CE 3D-GRE) imaging for detecting brain metastases, requiring additional scan time. In this study, we proposed deep-learned 3D BB imaging with an auto-labelling technique and 3D convolutional neural networks for brain metastases detection without additional BB scan. Patients were randomly selected for training (29 sets) and testing (36 sets). Two neuroradiologists independently evaluated deep-learned and original BB images, assessing the degree of blood vessel suppression and lesion conspicuity. Vessel signals were effectively suppressed in all patients. The figure of merits, which indicate the diagnostic performance of radiologists, were 0.9708 with deep-learned BB and 0.9437 with original BB imaging, suggesting that the deep-learned BB imaging is highly comparable to the original BB imaging (difference was not significant; *p* = 0.2142). In per patient analysis, sensitivities were 100% for both deep-learned and original BB imaging; however, the original BB imaging indicated false positive results for two patients. In per lesion analysis, sensitivities were 90.3% for deep-learned and 100% for original BB images. There were eight false positive lesions on the original BB imaging but only one on the deep-learned BB imaging. Deep-learned 3D BB imaging can be effective for brain metastases detection.

## Introduction

Brain metastases are the most common intracranial tumors and their prevalence has been increasing due to prolonged survival of cancer patients with improved systemic therapies^1^. Although the majority of patients with brain metastases have poor prognosis, those with a limited disease may have a more favorable outcome with intensive therapies. Various treatment options are available depending on the number, size and location of brain metastases, including surgery, stereotactic radiosurgery, whole-brain radiotherapy and chemotherapy^1,2^. In addition, the presence of brain metastases can alter the overall oncologic management; hence, an early and accurate diagnosis of metastatic lesions is crucial for appropriate treatment planning. Contrast-enhanced magnetic resonance imaging (MRI) is the imaging modality of choice for patients with suspected brain metastases.

**Figure 1:**
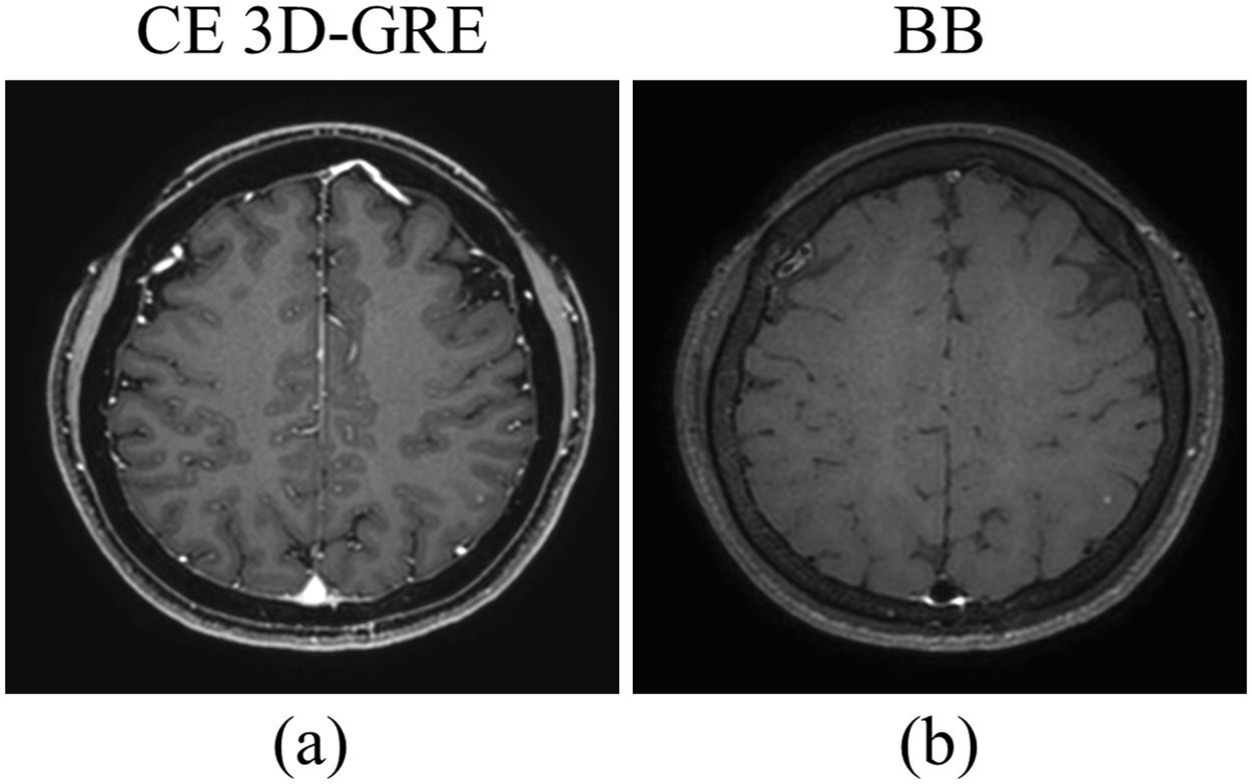
Example images of (**a**) contrast-enhanced 3D gradient-echo (CE 3D-GRE) and (**b**) black-blood (BB) imaging sequences. Several linear and dot-like enhanced blood vessels can be seen along the sulci, and it was difficult to delineate a tiny metastatic lesion among these enhanced vessels on the CE 3D-GRE imaging. Conversely, on the BB imaging, only the metastatic lesion in the left parietal lobe retained a high signal intensity.

Recently, contrast-enhanced 3D gradient-echo (CE 3D-GRE) imaging has been widely used to detect brain metastases, allowing the detection of tiny metastases with its high spatial resolution and low partial volume effects^3^. However, blood vessels as well as metastatic lesions demonstrate high signal intensity on CE 3D-GRE imaging. Because of this, normal blood vessels are occasionally mistaken for metastatic lesions, and the detection of metastatic lesions can be affected by small vessels, particularly when the lesion is close to the cortex (Fig. 1a). Distinguishing tiny metastatic lesions from numerous small blood vessels can demand much time and effort from radiologists.

Black-blood (BB) imaging uses various methods to selectively suppress vessel signals and has been reported to improve diagnostic performance in detecting brain metastases^4–6^. A tiny enhancing metastasis is more readily detected on BB imaging than on CE 3D-GRE (Fig. 1b). However, vessel signal suppression is not always perfect, even at a low velocity-encoding setting, which may result in false positive results. Therefore, BB imaging is used to complement CE 3D-GRE imaging, requiring an additional scan time of approximately 5–7 min to cover the entire brain.

In clinical practice, radiologists diagnose diseases using various medical images obtained from different modalities, such as computed tomography (CT), MRI and positron emission tomography. Several studies have generated or transformed medical images with different contrasts, such as estimating CT images from MRI data and generating synthetic MRI^7,8^. These could reduce the number of scans needed for accurate diagnosis, thereby reducing the acquisition time. Recently, studies have been proposed using convolutional neural networks (CNNs), a type of deep-learning architecture, to generate or transform medical images. In one study, CNNs were used for reconstruction of 7 T-like magnetic resonance (MR) images from 3T images^9^. Another study used CNNs to estimate CT images from MR images^10,11^. These studies involved feeding pairs of two images with different contrasts into CNNs to train them on non-linear relationships between the two images. CNNs trained in this way could then be used to obtain images from other images without the need for an additional scan. These studies demonstrated the feasibility of generating or transforming medical images with different contrasts using CNNs.

However, there can be a problem with using CNNs to directly transform CE 3D-GRE images into BB images because of the different contrast and quality of BB images. BB imaging is based on a fast spin-echo sequence, in which T2 contrast is affected by the need for a long echo time than that for CE 3D-GRE imaging; this results in lower contrast between the white matter (WM) and grey matter^12,13^. Furthermore, BB imaging requires a long scan time, which increases the risk of motion-related artifacts. Consequently, training CNNs for directly transforming CE 3D-GRE images into BB images becomes inefficient, and blur artifacts can occur in the output images, thereby obscuring metastatic lesions because of the quality and contrast difference between the input (CE 3D-GRE) and label (BB) images (Supplementary Fig. S1).

Addressing this label problem for BB images for the efficient training of CNNs requires a labelling technique that creates new label BB images (referred to here as ‘synthetic BB images’) from CE 3D-GRE images by removing blood vessels while retaining the metastases. If such a labelling process were manually implemented, then it would require trained radiologists to erase blood vessels by comparing CE 3D-GRE and BB images, which would be complex, labor intensive and time consuming. Therefore, we developed an auto-labelling technique that used the pre-acquired CE 3D-GRE and BB images to label synthetic BB images for CNNs, producing vessel-suppressed images that retained the metastases. Then, we propose a novel deep-learned 3D BB imaging that uses 3D CNNs trained with CE 3D-GRE and synthetic BB images. We validated the resulting deep-learned BB images by assessing the degree that the vessels were suppressed and the conspicuity of metastatic lesions.

In summary, we propose a deep-learned 3D BB imaging that uses 3D CNNs with an auto-labelling technique for detecting metastatic brain tumors. We demonstrate that this method can be useful for the accurate diagnosis of metastatic brain tumors, reducing MRI scan time, and that it has the potential to be applied to various forms of medical imaging.

## Methods

### Patient population

**Figure 2:**
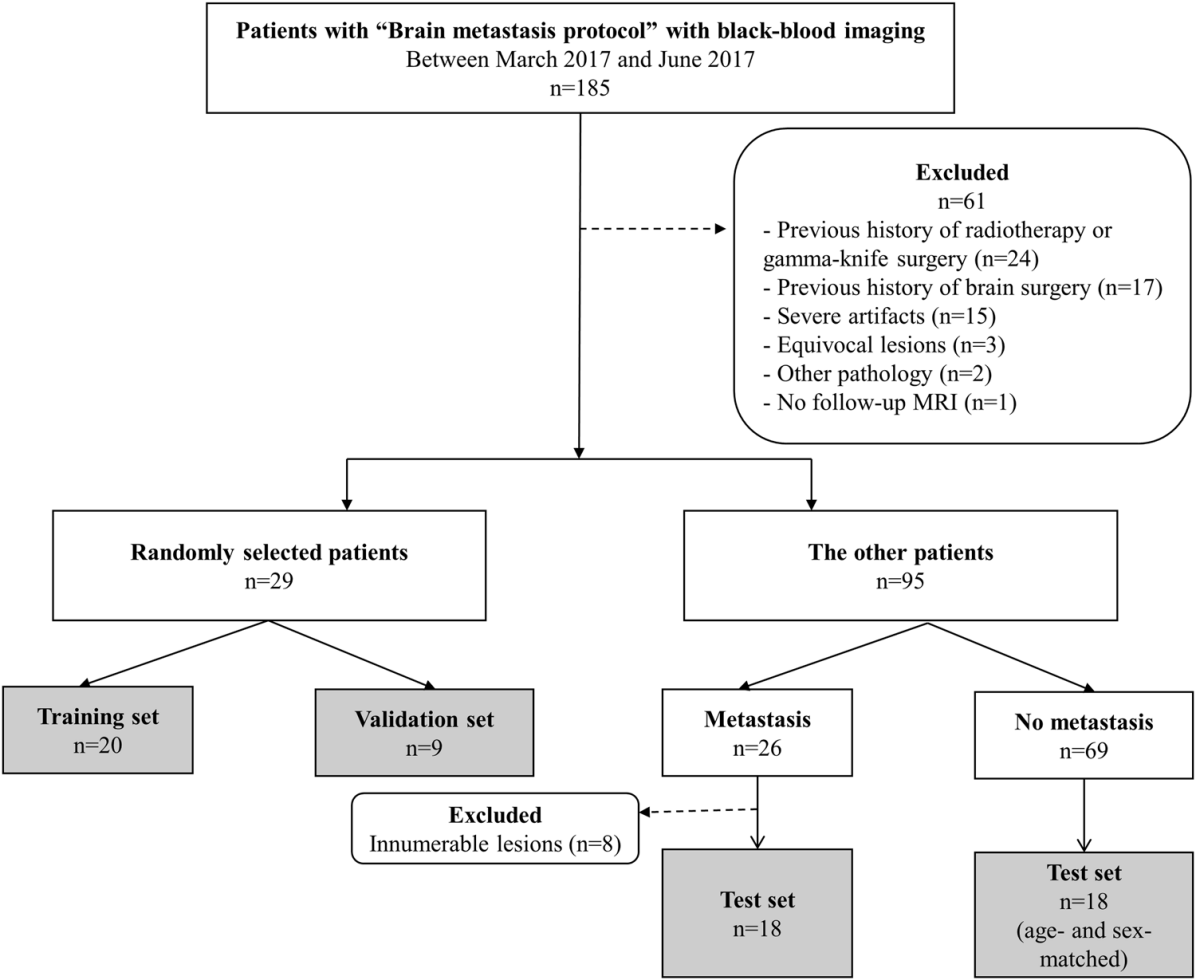
Flowchart of the patient population.

This retrospective study was approved by our Institutional Review Board, which waived the requirement to obtain written informed consent. Records for 185 consecutive MRI studies between March and June 2017 that followed our “Brain metastasis protocol” were retrieved from the radiology database. Of these, 61 patients were excluded for the following reasons: radiotherapy or gamma-knife surgery for brain metastases before undergoing MRI; a previous history of surgery; severe artifacts, equivocal lesions or other pathology or no follow-up MRI. A flowchart of the study population is shown in Fig. 2. Of the remaining 124 patients who underwent brain MRI for metastasis evaluation, 29 (men, 10 and women, 19; mean age, 60 ± 14 years) were randomly selected—20 patients for training set (11 patients with metastasis and 9 patients without metastasis) and 9 for validation set (6 patients with metastasis and 3 patients without metastasis). A further 18 patients with metastasis (men, 6 and women, 12; mean age, 56.9 ± 9.8 years) were selected as a test set and were included in qualitative and quantitative analyses; the primary cancer sites in these patients were the lungs for 15 patients and breast for 3. In addition, 18 patients without metastasis (men, 6 and women, 12; mean age, 57 ± 6.9 years) were chosen out of 69 after age and sex matching and were also included in the test set.

Metastatic brain tumors were determined by a review of both initial and follow-up MR images. Briefly, enhancing lesions that newly appeared or those that increased in size on the follow-up MRI, or lesions that decreased in size after radiation therapy or chemotherapy, were considered metastatic lesions.

### Image acquisition

MRI was performed using a 3.0-T MRI scanner (Ingenia CX, Philips Medical Systems, Best, The Netherlands) with an eight-channel sensitivity-encoding head coil. Five minutes after administering a gadolinium-based contrast (0.1 ml/kg gadobutrol; Gadovist, Bayer Schering Pharma), 3D-GRE imaging was performed followed by a 3D fast spin-echo sequence with the improved motion-sensitized driven-equilibrium (BB imaging). The imaging parameters for CE 3D-GRE imaging were as follows: repetition time (TR)/echo time (TE), 8.6/4.7 ms; flip angle, 8°; field of view, 20–24 cm; section thickness, 1 mm; matrix, 240 × 240 and acquisition time, 4 min 32 s. The parameters for the fast spin-echo sequence were as follows: TR/TE, 500/28.9 ms; flip angle, 90°, field of view, 20–24 cm; section thickness, 1 mm; matrix, 240 × 240 and acquisition time, 5 min 54 s. The improved motion-sensitized driven-equilibrium pre-pulse comprised one 90° excitation pulse, two 180° refocusing pulses and one 90° excitation pulse with motion-sensitized gradients between radiofrequency pulses. The duration between the two 90° pulses (TEprep) was 28.3 ms, and the flow velocity encoding for gradient pulses was 1.3 cm/s.

### Proposed deep-learned 3D BB imaging

**Figure 3:**
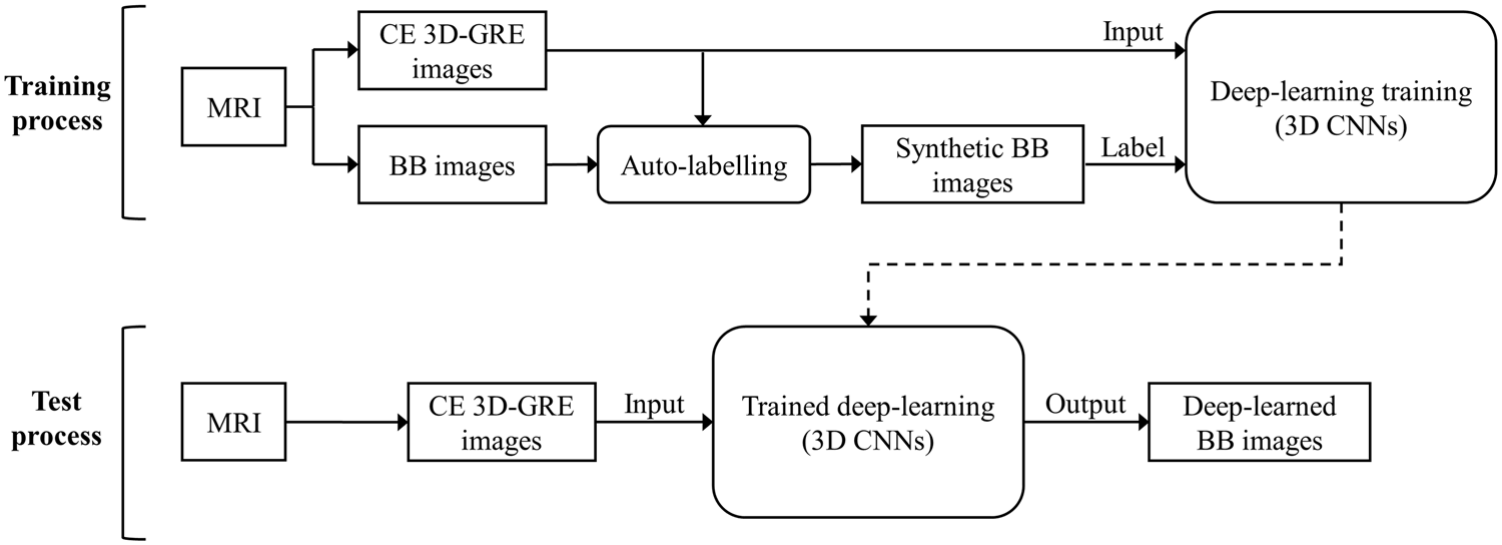
Flowchart of the deep-learned 3D black-blood (BB) imaging process, comprising training and test processes. In the training process, 3D convolutional neural networks (CNNs) were trained using input contrast-enhanced 3D gradient-echo (CE 3D-GRE) and label (synthetic BB) images. Labels were generated by an auto-labelling technique using pre-acquired CE 3D-GRE and BB images. In the test process, trained 3D CNNs were used for generating deep-learned BB images from CE 3D-GRE images.

Figure 3 shows a flowchart of the proposed deep-learned 3D BB imaging process, which is divided into 1) a training process for the deep-learning model and 2) a test process for obtaining results using the trained model. In the training process, 3D CNNs were used for the deep-learning model and trained by input (CE 3D-GRE images) and label (synthetic BB images generated by our auto-labelling method). For the test process, new CE 3D-GRE images were input into trained 3D CNNs, which produced deep-learned BB images as the output.

### Auto-labelling technique

The auto-labelling process comprised two parts: pre-processing and vessel extraction. Pre-processing included registration, intensity normalization and brain extraction. The registration process involved registering the BB image with reference to the CE 3D-GRE image using the ‘Optimized Automatic Image Registration 3D’ algorithm included in MIPAV version 7.4.0 software^14^. For intensity normalization, each pixel of 3D volume images was divided by the maximum pixel value in that volume images, resulting in an intensity range of 0–1. The brain extraction process extracted only the brain tissue from the CE 3D-GRE image using the ‘Brain Extraction Tool’ algorithm in FSL software^15,16^.

**Figure 4:**
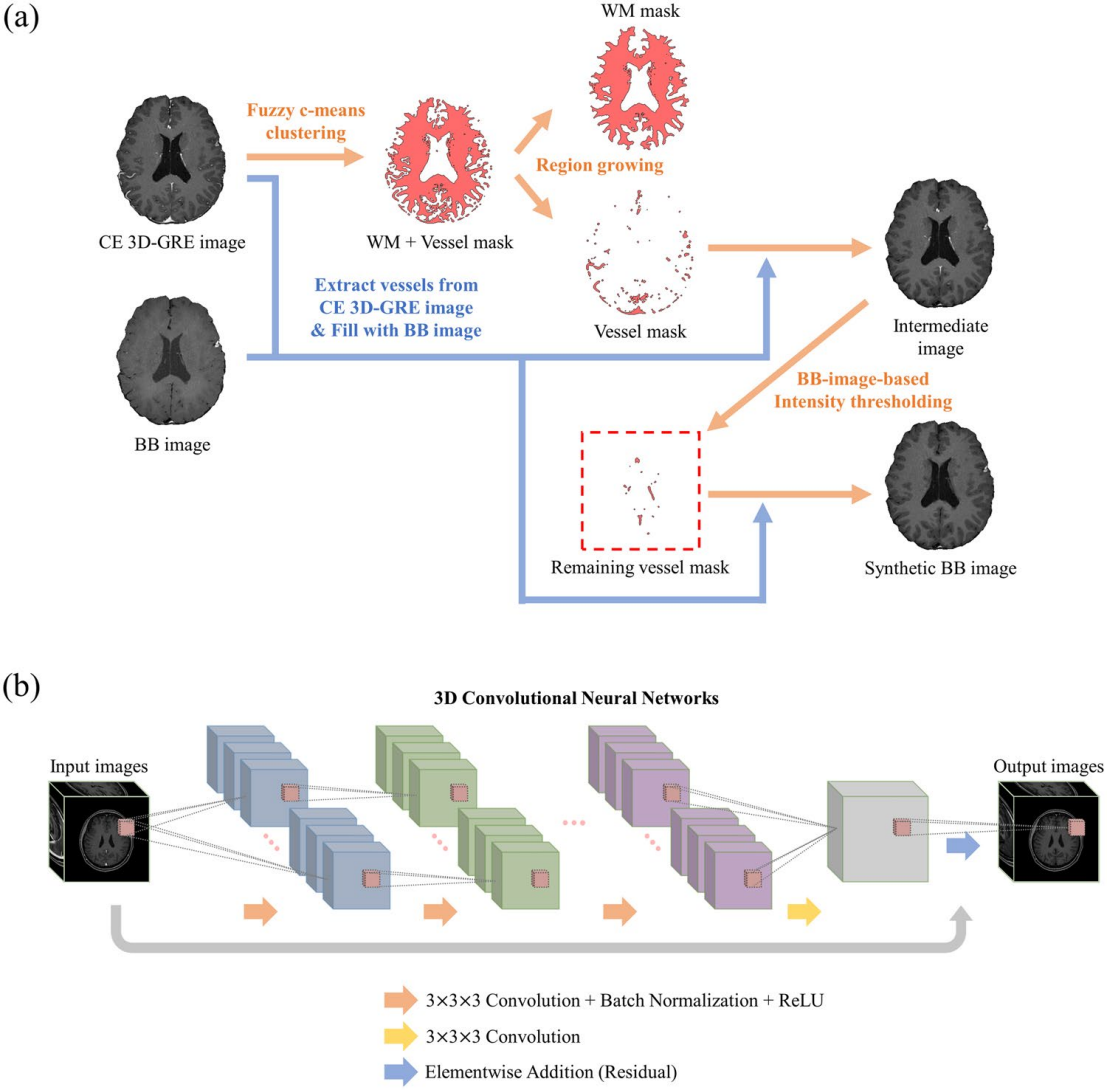
(**a**) The vessel extraction process of the auto-labelling technique. First, the vessel mask was generated using fuzzy c-means clustering and region growing methods from the contrast-enhanced 3D gradient-echo (CE 3D-GRE) image. Blood vessels were extracted using the generated vessel mask and then filled with the corresponding pixels from the black-blood (BB) image. The remaining blood vessels were extracted using a second vessel mask generated by BB-image-based intensity thresholding and filled with the corresponding pixels from the BB image. After vessel extraction, synthetic BB image is made. (**b**) The deep-learning architecture based on 3D convolutional neural networks (CNNs). CE 3D-GRE images formed the input of 3D CNNs, with deep-learned BB images as the output.

The vessel extraction process extracted blood vessels from a CE 3D-GRE image using CE 3D-GRE and BB images without the skull obtained during the pre-processing step. Figure 4a shows the process in detail. Using the fuzzy c-means clustering method^17^, WM and vessel mask were together generated from the CE 3D-GRE image, and the region growing method^18^ was then used to separate out only vessel mask. Blood vessels were extracted from the CE 3D-GRE image using the vessel mask created as described above, and areas corresponding to the vessel mask were filled with the corresponding parts of the paired BB image, replacing vessel signals in the CE 3D-GRE image with suppressed signals in the BB image. During this filling process, signal intensity near the edges of filled areas discretely changes, which can result in a heterogeneity in the synthetic BB image and consequently result in an inefficient training of CNNs. To address this, areas peripheral to the vessel mask were dilated and filled with the weighted summation of the corresponding CE 3D-GRE and BB image pixels. Although it was possible to create an intermediate synthetic BB image at this stage, blood vessels remained in several parts of the image. To remove the remaining blood vessels, the intermediate synthetic BB image was subjected to BB-image-based intensity thresholding. Conventional intensity thresholding would have extracted not only blood vessels but also any areas of WM with the same or higher intensity value as blood vessels. To resolve this, we made a difference image of the intermediate synthetic BB image and the BB image to reduce the signal intensity of all areas apart from blood vessels. With the help of BB images, the intensities of non-vessel tissue areas were considerably reduced, allowing the remaining blood vessels in the intermediate image to be effectively extracted through intensity thresholding. After the second vessel extraction process, areas corresponding to the vessel mask were filled with the corresponding BB image pixels and areas peripheral to the vessel mask were dilated and filled with the weighted summation of the intermediate synthetic BB image and BB image pixels. This resulted in the synthetic BB image.

### Deep-learning architecture (3D CNNs)

The deep-learning architecture is shown in Fig. 4b. This used 3D CNNs for learning the non-linear relationship between the input and output. The architecture comprised 25 3D convolution layers, with a residual learning structure for the elementwise summation of the input to the output of the final convolution layer. The kernel size of each 3D convolution layer was 3 × 3 × 3 and the number of feature maps was 64, except in the final convolution layer. After each convolution layer, batch normalization^19^ was performed using a rectified linear unit (ReLU)^20^ as the activation function. The output size of each convolution layer was kept the same by padding the input to the layer with one pixel at the top, bottom, left and right sides. The kernel size of the final convolution layer was also 3 × 3 × 3 and the number of feature maps was set to one to make the final output size equal to the input. The convolution layer weights were initialized with MSRA initialization^21^. These parameters were updated by backpropagation using the Adam optimizer^22^. We adopted the residual learning architecture by implementing elementwise addition after the final convolution layer, which enabled fast training^23,24^. The learning rate was 1 × 10^−5^ and decreased by a factor of 10 every 10 epochs. Mean squared error was used to calculate the loss between the output and label. We clipped the gradient by 1, which prevented gradient explosion^24^. The batch size for the training and testing was 10. For training, we made the input and output images into patches sized 31 × 31 × 31. These patches were selected from the same location of volume images because the input and output images had been registered during the pre-processing. For testing, volume images were fed into the trained network as the input.

Data from 20 patients were used for training, 9 for the validation and 36 as test data. Training was stopped at the saturation point at which the validation loss did not decrease, and a total of 30 epochs of learning were performed. Subsequently, a fine-tuning process was performed using the validation data of three patients who had small metastatic lesions, with an initial learning rate that was 1/10 that used in the training process but with all other conditions identical. A total of 60 epochs of fine-tuning were performed until the saturation point at which the validation loss did not decrease. The deep-learning architecture was implemented by using the deep-learning library Tensorflow^25^. The training and fine-tuning of 3D CNNs took approximately 3 d. The test for reconstructing output volume images took approximately 1 min using an Intel Xeon E5-1650 CPU with a Nvidia Titan X GPU and 64 GB RAM.

### Quantitative image analysis

To evaluate the efficiency of blood vessel suppression in the deep-learned BB images, we measured the ratio of WM to vessel signals. The vessel signal was measured by selecting nine regions of interest (ROIs) in a single axial image at the level of the thalamus. Blood vessels were classified into three types. Three ROIs (type 1) were selected from the anterior cerebral artery, superior sagittal sinus and vein of Galen; a further three ROIs (type 2) were selected from the middle cerebral arteries, and the remaining three ROIs (type 3) were selected from among the small cortical branches (Supplementary Fig. S2). ROI for WM was selected from a homogeneous area in the centrum semiovale. We defined the vessel suppression ratio *R*_*s*_ as follows:

$${{R}}_{s}=\frac{{S}{{I}}_{{WM}}}{{0.5}({S}{{I}}_{{WM}}+{S}{{I}}_{{vessel}})}$$

where *SI*_*WM*_ is the mean signal for WM ROI, and *SI*_*vessel*_ is the mean signal for three vessel ROIs of one vessel type. The ratio *R*_*s*_ ranges from 0 to 2, with values approaching 2 indicating that the vessel signal is completely suppressed similar to the signal intensity of the background. Thus, higher values of the ratio *R*_*s*_ indicate that vessel suppression is effective while WM signal is preserved.

### Qualitative image analysis

One neuroradiologist (S.S.A., with 8 years of experience in neuroradiology) reviewed the image quality of synthetic BB in terms of vessel suppression and lesion conspicuity in the training set. The reviewer recorded the degree of vessel suppression on a three-point scale: 1, poor (>5 cortical vessels visualised); 2, fair (3–5 cortical vessels visualised) or 3, excellent (<3 cortical vessels visualised) and also rated the lesion conspicuity, if present, on a three-point scale: 1, poor (barely seen); 2, fair or 3, excellent.

Two neuroradiologists who were blinded to the clinical history and pathology (S.H.B. and Y.W.P., each with 2 years of experience in radiology) independently evaluated the original and deep-learned BB imaging in the test set at sessions 2 weeks apart. All images were reviewed in a random order during each session. Each reviewer recorded the degree of vessel suppression in the deep-learned BB imaging on a three-point scale: 1, poor (>5 cortical vessels visualised); 2, fair (3–5 cortical vessels visualised) or 3, excellent (<3 cortical vessels visualised). They also rated the lesion conspicuity on the original BB and deep-learned BB images, again on a three-point scale: 1, poor (barely seen); 2, fair or 3, excellent. Another neuroradiologist (S.S.A., with 8 years of experience in neuroradiology) combined the results from the two reviewers for original and deep-learned BB images. The interobserver agreement for the visual grading was assessed using weighted kappa statistics^26^. Kappa values can be interpreted as follows: 0.81–1.0, excellent agreement; 0.61–0.80, good agreement; 0.41–0.6, moderate agreement; 0.21–0.4 fair agreement and 0–0.2 only slight agreement. Lesions with conspicuity scores of 2 or 3 points were considered positive for metastasis. Each lesion was classified according to its maximum diameter in the axial imaging plane as either <2 or ≥2 mm.

### Statistical analysis

Paired t-tests^27^ were used to compare suppression ratios of CE 3D-GRE, original BB and deep-learned BB images according to the three vessel types. These tests were performed using Excel software (Microsoft Corp., Redmond, WA), with *p*-values < 0.05 considered statistically significant. The diagnostic accuracy of radiologists using deep-learned or original BB images was evaluated using a jackknife free-response receiver operating characteristic (JAFROC) analysis^28,29^. This method is used to statistically compare radiologists’ diagnostic performance in different modalities. We used JAFROC version 4.2.1 software (http://www.devchakraborty.com) to calculate the figure of merit values.

### Data availability

The datasets generated during and/or analyzed during the current study are not publicly available due to the confidential nature of the clinical imaging data and potential risk of personal information leakage but can be made available from the corresponding authors on reasonable request.

## Results

With the proposed auto-labelling technique and deep-learning model (3D CNNs), synthetic BB images were effectively generated for the training, and final deep-learned BB images were successfully inferred in all of the test set. In the evaluation of synthetic BB images in the training set, blood vessel signals were effectively suppressed in all patients (with 16 patients showing excellent suppression and four showing fair suppression). The average score of vessel suppression was 2.8 (excellent). Among 11 patients with metastatic lesions, all metastatic lesions remained in the synthetic BB and overall lesion conspicuity was excellent in eight patients and fair in three patients (Supplementary Fig. S3). The average score of lesion conspicuity was 2.7 (excellent). Both in the per patient analysis and per lesion analysis, sensitivities were 100% for synthetic BB imaging.

**Figure 5:**
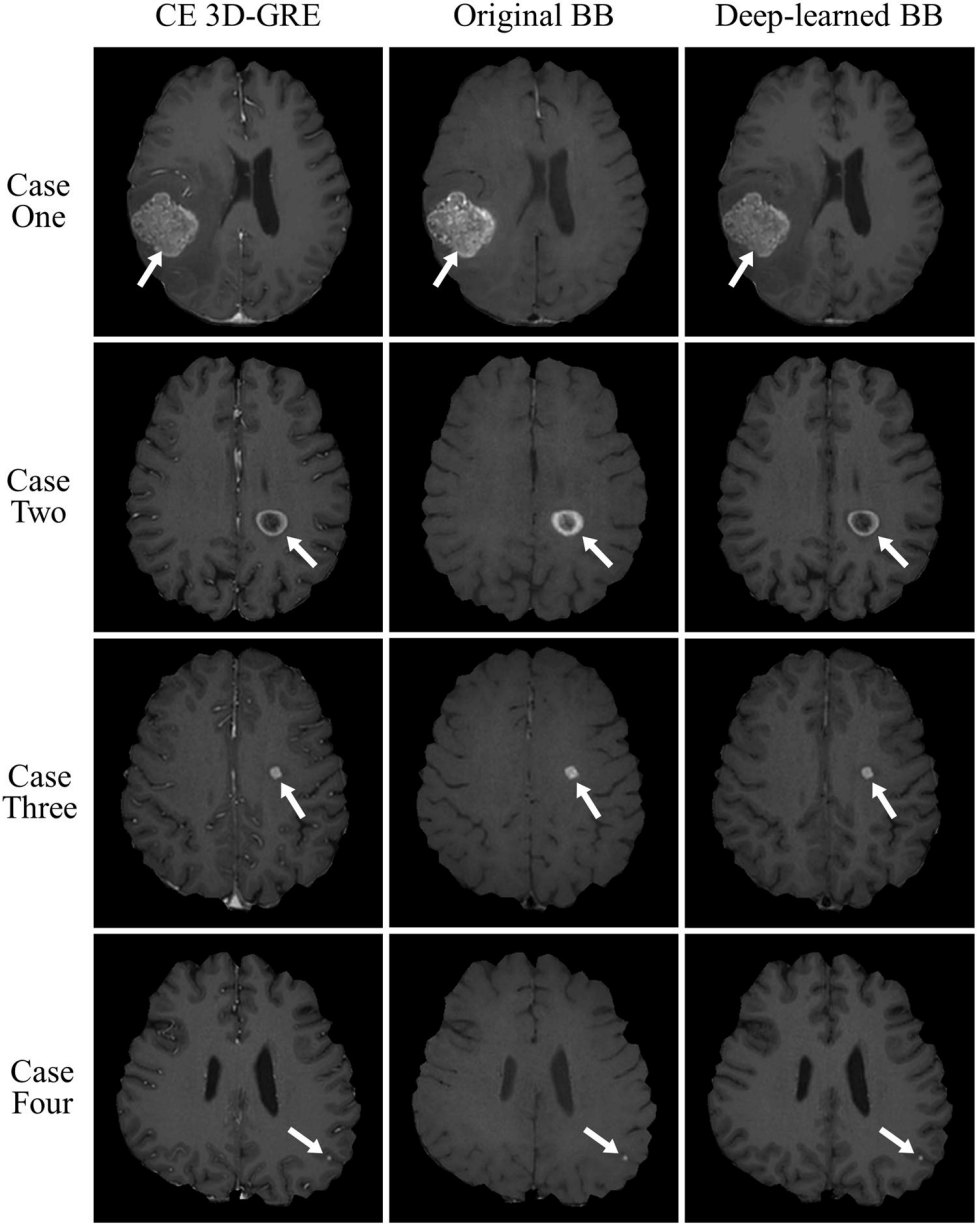
True positive results from original and deep-learned black-blood (BB) images. Each column represents (from left to right) a contrast-enhanced 3D gradient-echo (CE 3D-GRE) image, an original BB image and a deep-learned BB image produced by the deep-learning process. The rows represent different patients with metastases of different sizes. In all cases, metastases are well observed on both original and deep-learned BB images.

Figure 5 shows example CE 3D-GRE, original BB and deep-learned BB images for four cases with metastasis sizes of 30 mm, 15 mm, 6 mm and 3 mm. All metastases at all these different sizes were well observed in both original and deep-learned BB images. As seen in the third column, blood vessels were well suppressed and metastases of all sizes were retained in the deep-learned BB images. In particular, the 3-mm metastasis in the last case, located in the grey matter close to the cortex in the CE 3D-GRE image, remained in the deep-learned BB image although its size and shape were similar to those of small cortical vessels.

**Figure 6:**
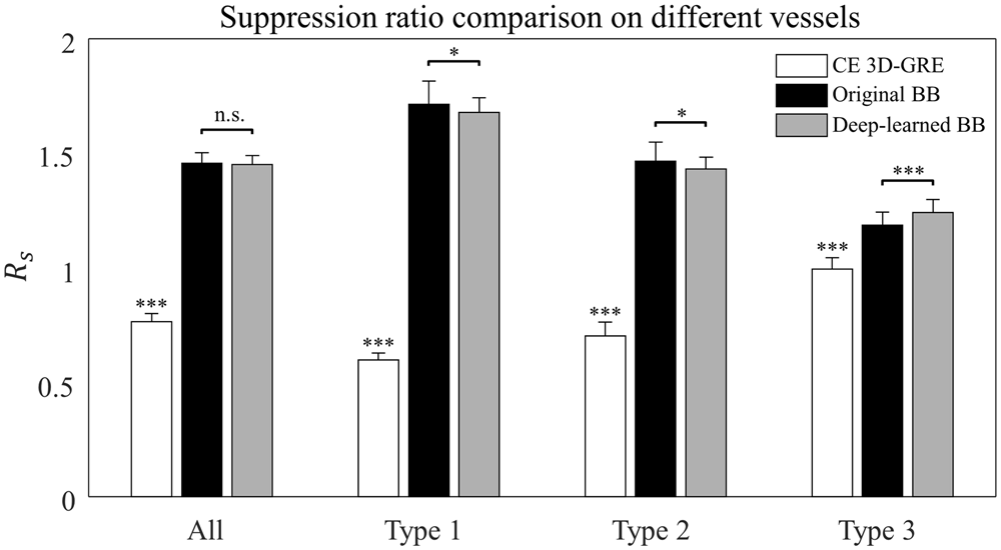
Vessel suppression ratio comparison for different vessels in contrast-enhanced 3D gradient-echo (CE 3D-GRE) images, original black-blood (BB) images and deep-learned BB images of the 36 test patients. Paired t-tests were used to calculate *p*-values. *p < 0.05, ***p < 0.001, n.s, not significant (*p* > 0.05).

Figure 6 shows the vessel suppression ratio *R*_*s*_ comparison for different vessels among the CE 3D-GRE, original BB and deep-learned BB images for all 36 patients. Original and deep-learned BB images both had significantly higher ratios than CE 3D-GRE images across all vessel types (1.455 vs. 0.765, *p* < 0.001 for original BB and CE 3D-GRE images and 1.450 vs. 0.765, *p* < 0.001 for deep-learned BB and CE 3D-GRE images). For type 1 and type 2 vessels, original BB images had a higher ratio than deep-learned BB images (type 1: 1.715 vs. 1.678, *p* < 0.05 and type 2: 1.464 vs. 1.431, *p* < 0.05). However, for type 3 vessels, deep-learned BB images had a significantly higher ratio than original BB images (1.241 vs. 1.186, *p* < 0.001). Overall, original and deep-learned BB images showed no statistical difference in the vessel suppression ratio (1.455 vs. 1.450, *p* > 0.05).

**Figure 7:**
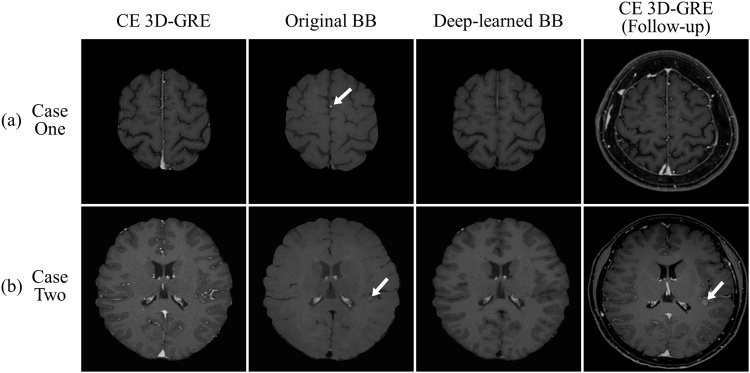
(**a**) False positive results from original black-blood (BB) images and (**b**) false negative results from deep-learned BB images. In case 1, the original BB image showed a high signal intensity (arrow) in the left frontal lobe that mimicked a metastasis but which was due to incomplete vessel suppression in the sulcus, as seen on the contrast-enhanced 3D gradient-echo (CE 3D-GRE) image. The deep-learned BB image showed no abnormality in the corresponding area. There is still no metastatic lesion suspected on follow-up scan after 4 months. In case 2, the original BB image demonstrates a tiny high signal intensity lesion (arrow) in the left temporal lobe, which was not seen on the deep-learned BB image. Before follow-up, radiologists could not identify the lesion on the CE 3D-GRE image because of adjacent cortical vessels, which may explain why deep-learned BB failed to show the lesion. The lesion became evident with a follow-up scan after 2 months and was confirmed as a metastasis.

Interobserver agreements for vessel suppression were good to excellent (kappa values, 0.801 for deep-learned BB images and 0.636 for original BB images). Interobserver agreements for lesion conspicuity were good to excellent (kappa values, 0.788 for deep-learned BB images and 0.878 for original BB images). The test set included 18 patients with metastatic brain tumors. In total, they had 62 tumors, with a median maximum diameter of 4.1 mm (interquartile range, 2.0–6.8 mm); 50 of the lesions were ≥2 mm but only seven were >10 mm. On deep-learned BB imaging, blood vessel signals were effectively suppressed in all patients (with 34 patients showing excellent suppression and two showing fair suppression). In the per patient analysis, sensitivities were 100% for both deep-learned and original BB imaging; however, the original BB imaging indicated false positive results for two patients (Table 1) (Fig. 7a). In the per lesion analysis, the overall sensitivities were 90.3% for deep-learned BB imaging and 100% for the original BB imaging. In subgroup analysis for lesions ≥2 mm, deep-learned BB imaging missed only one lesion, with 98% sensitivity. Eight false positive lesions were seen on the original BB imaging, whereas only one was seen on the deep-learned BB imaging. In the JAFROC analysis, the figure of merit values were 0.9708 with deep-learned BB imaging and 0.9437 with original BB imaging, a difference that was not significant (*p* > 0.05).

**Table 1:** Comparison of metastatic lesion detection performances of deep-learned and original black-blood imaging.

	Deep-learned black-blood	Original black-blood
**Per patient**		
Sensitivity	100% (18/18)	100% (18/18)
False positive	1	2
**Per lesion**		
Sensitivity	90.3% (56/62)	100% (62/62)
Sensitivity (≥2 mm)	98% (49/50)	100% (50/50)
False positive	1	8
**Figure of merit**	0.9708	0.9437

## Discussion

BB imaging is often used as a complementary modality to CE 3D-GRE imaging for brain metastasis diagnosis, but in routine clinical settings, this requires an additional scan. With our specialized deep-learning technique, we could effectively suppress blood vessel signals, transforming CE 3D-GRE images into deep-learned BB images, thereby obtaining good diagnostic results in terms of lesion detection (figure of merit value, 0.9708), without the need to acquire additional BB images. The original BB imaging was sufficiently sensitive to readily detect even lesions <2 mm (with 100% sensitivity); however, there were false positives, where incomplete blood vessel suppression mimicked metastatic lesions. Although modern BB imaging has been much improved by advanced techniques such as improved motion-sensitized driven-equilibrium technique^6,30^, the signal suppression of blood vessels is not yet perfect, leaving some blood vessels bright on BB images, which can result in false positive lesion detection; hence, CE 3D-GRE imaging is usually referred for confirming whether the identified lesions are actual metastatic lesions or just blood vessels.

The objective of our method was to suppress blood vessels in CE 3D-GRE images while retaining the metastases. This can be considered a method for extracting blood vessels from medical images; therefore, differences between this method and conventional vessel segmentation or extraction methods should be considered. First, most conventional vessel extraction methods were developed for blood vessel images, such as retinal images and angiography. To the best of our knowledge, no study has reported on extracting blood vessels from CE 3D-GRE MR images. There have been several studies of vessel segmentation using Hessian-based, region growing and geometric flow methods, which take into consideration 3D structures and flow characteristics of blood vessels^31–34^. These methods were developed for vessel-specific images in which vessel signals showed high contrast compared with other tissues or the background. They relied on the connectivity of blood vessels that branch off from the main vessels. However, small blood vessels, such as cortical branches, sometimes have lower signal intensities than WM on CE 3D-GRE images, and their connections to the main vessels may not be evident on axial images (Fig. 1a). Therefore, it is difficult to apply conventional vessel extraction or segmentation methods, developed for retinal and angiography images, to CE 3D-GRE images. However, our method suppressed blood vessels quite well in CE-GRE images while retaining the metastases, and most resulting deep-learned BB images were evaluated as excellent by the two radiologists. In addition, the vessel suppression ratios *R*_*s*_ of deep-learned and original BB images (Fig. 6) did not significantly differ across all vessel types (*p* > 0.05), indicating that blood vessels were well suppressed in deep-learned BB images, just as in original BB images. Notably, for the type 3 vessels, which were selected from small cortical branches, deep-learned BB images had a higher vessel suppression ratio *R*_*s*_ than original BB images (*p* < 0.001).

Our method not only suppressed blood vessels but also retained the metastases. CE 3D-GRE images included several small metastases with blob shapes and diameters similar to those of cortical branches (Fig. 5). Conventional vessel extraction methods were not developed to distinguish brain metastases from blood vessels; hence, if they were applied to CE 3D-GRE images for vessel extraction, then it is likely that small metastases would be extracted together with blood vessels. To test this, we applied a 3D model-based vascular segmentation method^34^ to extract blood vessels from CE 3D-GRE images and observed that several metastatic lesions were indeed extracted together with blood vessels (Supplementary Fig. S4). In contrast, our method showed high sensitivity (90.3%) and excellent performance, particularly in lesions ≥2 mm, with 98% sensitivity in the per lesion analysis (Table 1) and only one false positive case in deep-learned BB images. Even some tiny lesions (<2 mm) were well observed on deep-learned BB images (Supplementary Fig. S5). Furthermore, the interobserver agreement for lesion conspicuity was good (kappa value, 0.788). These results suggest that our deep-learning-based method can distinguish metastases from blood vessels and that it can be used for the accurate diagnosis of brain metastases.

Several studies investigated the sensitivity of CE 3D-GRE images for detection of small lesions (<5 mm) and reported sensitivities ranging from 45.9% to 71.9%^4,5^, which suggested that detecting tiny lesions from CE 3D-GRE images was difficult even for radiologists. Another recent study investigated learning techniques to detect metastatic lesions from CE 3D-GRE images and reported 73.7% sensitivity for tiny lesions (<2 mm) at the cost of a large number of false positives (302 false positive cases per subject)^35^. In contrast, our proposed deep-learned BB images which were generated from CE 3D-GRE images using the trained deep-learning model resulted in 58.3% and 83.3% sensitivities in subgroup analysis for lesions <2 mm and <5 mm, respectively, with only one false positive lesion in the test set (Table 1) (Supplementary Fig. S5). However, several lesions <2 mm were missed, which may have been misidentified as blood vessels and their signal may have been suppressed by 3D CNNs in the same manner as for blood vessels (Fig. 7b). Further study with a larger population size is needed to improve sensitivity for tiny lesions.

The purpose of the auto-labelling technique was to make new labels (synthetic BB images) for the efficient learning of CNNs. Because of the discrepancy in contrast and quality between CE 3D-GRE and BB images, BB images were inappropriate for labels of CNNs in a way that would allow CE 3D-GRE images to be directly transformed into BB images. This meant that the training of CNNs might be inefficient and that blur artifacts might occur. Usually, the L2 distance loss function is used in CNNs on the assumption that distributions of input and label data are Gaussian^36^. However, with a multimodal distribution, this can result in blur artifacts in the CNNs’ output. We tried training CNNs to transform CE 3D-GRE directly into BB images and observed the occurrence of blur artifacts and that metastatic lesions were not retained (Supplementary Fig. S1). To overcome these problems, we developed the auto-labelling technique to make a new label (synthetic BB image) that suppressed vessels and retained metastases while leaving other tissue contrasts unchanged. This technique extracted blood vessels from CE 3D-GRE images using fuzzy c-means clustering, region growing and intensity thresholding methods. Parts of blood vessels were not extracted by the fuzzy c-means clustering and region growing methods, but were completely extracted with our BB-image-based intensity thresholding method. Depending on the threshold value, the extracted areas corresponding blood vessels may change which results in different synthetic BB images. The optimal value of the threshold value may be slightly different depending on subjects; however, various threshold values in specific range worked well in our datasets, which resulted in good synthetic BB images. Thus, synthetic BB images were robustly generated for the training set using the auto-labelling technique (Supplementary Fig. S6). Using BB images, it was possible to produce images (synthetic BB images) in which vessel suppression was complete while other tissues and metastases were retained; this enabled efficient learning to generate deep-learned BB images from CE 3D-GRE images.

During the auto-labelling process, areas corresponding to the vessel mask on CE 3D-GRE image were filled with the corresponding BB image pixels. Thus, if there were false positive lesions (non-suppressed blood vessels) on the original BB image (as shown Fig. 7a), blood vessels might have been remained in the synthetic BB image as well. Nevertheless, we were able to generate deep-learned BB images that successfully suppressed vessel signals, which resulted in only one false positive in the test set (Table 1), using the trained deep-learning model (3D CNNs). Recent studies suggested that deep-learning is robust to noisy labels, i.e. incorrect labels, in large training data set^37,38^. Even if some blood vessels remained in the synthetic BB images, 3D CNNs were trained effectively in large training set to suppress blood vessels in CE 3D-GRE images while retaining the metastases (Supplementary Fig. S7). Thus, deep-learned BB images were successfully generated using the deep-learning model.

The auto-labelling concept and technique can be applied to other types of medical imaging transformation or generation, particularly those with multiple contrast images. The process is fully automatic, with no need for manual labelling. Most deep-learning studies require a sufficient quantity of labelled data; for example, one study of image translation by deep-learning used from 400 to >100,000 images as the training data set^39^. However, it is often difficult to acquire a sufficiently large amount of labelled data for deep-learning medical image applications because manual labelling is time consuming and the clinicians’ workload is considerable. The auto-labelling technique described here can be used as a quick and easy labelling method when applying deep-learning techniques to medical images.

Other studies investigated learning techniques to detect metastatic lesions fully or semi-automatically instead of by generating complementary BB images. One recent study used a machine learning technique for detecting brain metastases from MR images^35^. This study showed 87.3% sensitivity in detecting brain metastases, but reported 302 false positive cases per subject. Another study used deep CNNs to detect and segment brain metastases on MR images^40^. This study showed 86.1% sensitivity and reported 83 false positive cases per subject. Both studies focused on detecting brain metastases from MR images using neural networks. In contrast, our study focused on generating vessel-suppressed images (deep-learned BB) while retaining the metastases from CE 3D-GRE images by deep-learning (3D CNNs) without an additional MR scan. Another unique contribution of our study is that the auto-labelling technique has been developed and used effectively for the learning process, which obviates the need for extensive manual labelling work. In other studies^35,40^, they had to manually label all images for the training of neural networks.

Our study had several limitations. First, most diagnoses of metastasis were based on radiologic findings without pathologic confirmation because multiple tiny metastatic lesions are usually not resected in clinical practice. Thus, we cannot completely eliminate the possibility of false positives; however, we thoroughly reviewed both initial and follow-up MRI and excluded equivocal lesions. Second, the images were all obtained using the same MR scanner and with the same acquisition protocols for efficient training. Therefore, we may need to validate or optimize our trained 3D CNNs for images obtained using other MR scanners or different acquisition protocols. Third, for efficient training, we included only the supratentorial brain in the analysis; further study is needed to see if the method described can be applied to the posterior fossa.

In conclusion, deep-learned 3D BB imaging using an automatic labelling technique and 3D CNNs can be effectively used for detecting metastatic brain tumors.

## Electronic supplementary material

Supplementary Information
