# Supplementary material for: Deep-learned 3D black-blood imaging using automatic labelling technique and 3D convolutional neural networks for detecting metastatic brain tumors
Source: Sci Rep. 2018 Jun 21;8:9450. doi: 10.1038/s41598-018-27742-1 (PMC6013490; doi:10.1038/s41598-018-27742-1)
Supplement: Supplementary file 1 — Supplementary Information [file 41598_2018_27742_MOESM1_ESM.doc]

**Supplementary Information**

Deep-learned 3D black-blood imaging using automatic labelling technique and 3D convolutional neural networks for detecting metastatic brain tumors

Yohan Jun^1^, Taejoon Eo^1^, Taeseong Kim^1^, Hyungseob Shin^1^, Dosik Hwang^1,*^, So Hi Bae^2^, Yae Won Park^3^, Ho-Joon Lee^2^, Byoung Wook Choi^2^, and Sung Soo Ahn^2,*^

^1^School of Electrical and Electronic Engineering, Yonsei University, Seoul, Korea

^2^Department of Radiology and Research Institute of Radiological Science, Yonsei University College of Medicine, Seoul, Korea

^3^Department of Radiology, Ewha Womans University College of Medicine, Seoul, Korea

^*^corresponding authors: [dosik.hwang@yonsei.ac.kr](../../../../C:/Users/biosi/AppData/Local/Microsoft/Windows/Temporary%20Internet%20Files/Content.Outlook/XC7KD18H/dosik.hwang@yonsei.ac.kr) and [sungsoo@yuhs.ac](../../../../C:/Users/biosi/AppData/Local/Microsoft/Windows/Temporary%20Internet%20Files/Content.Outlook/XC7KD18H/sungsoo@yuhs.ac)


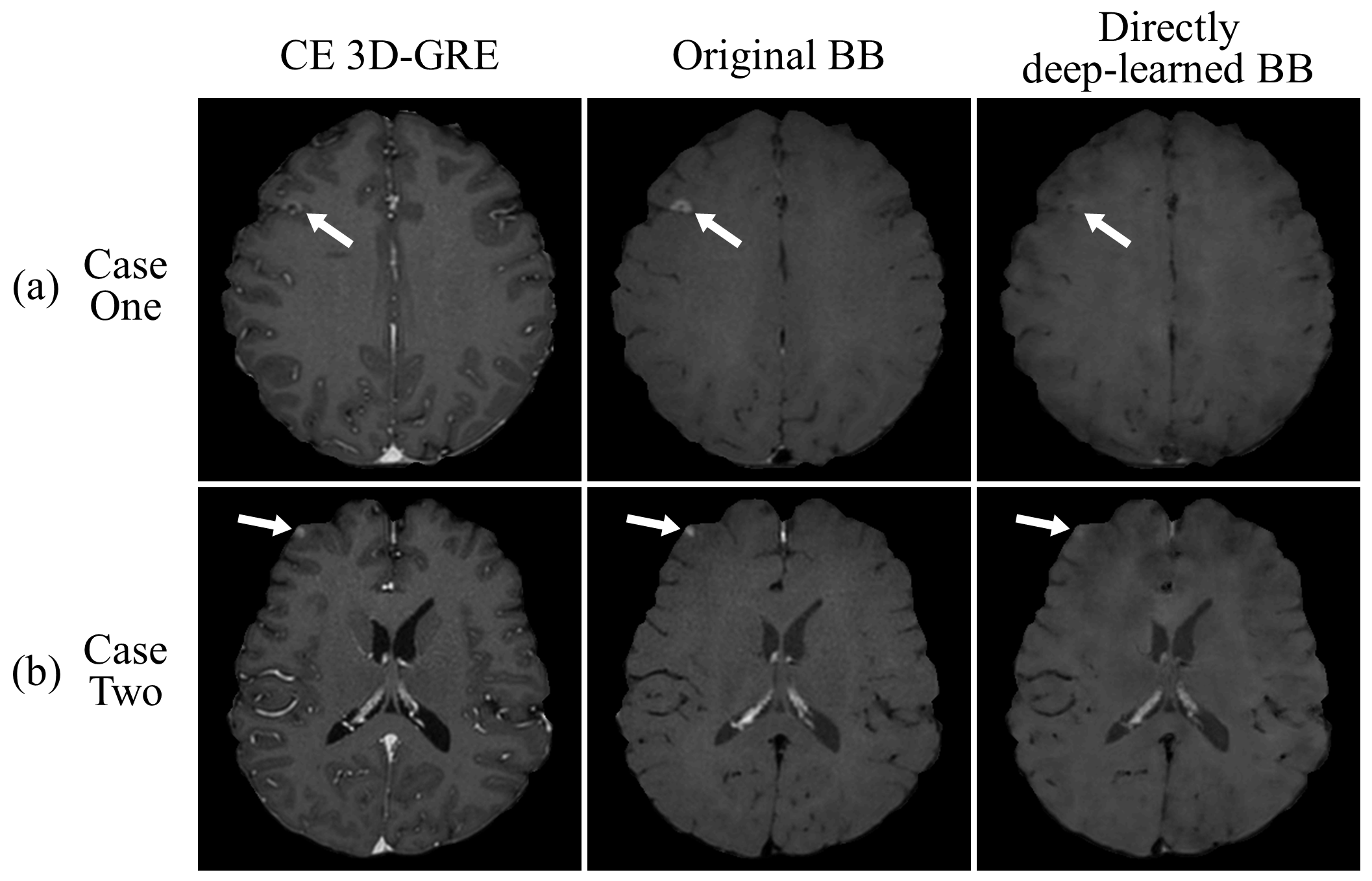


**Supplementary Figure S1**. Results of direct transforming CE 3D-GRE into BB images using 3D CNNs. In both case one and two, CE 3D-GRE and original BB images demonstrate high signal intense lesions in the right frontal lobe, which disappeared on directly deep-learned BB images due to severe blurring artifacts.

**
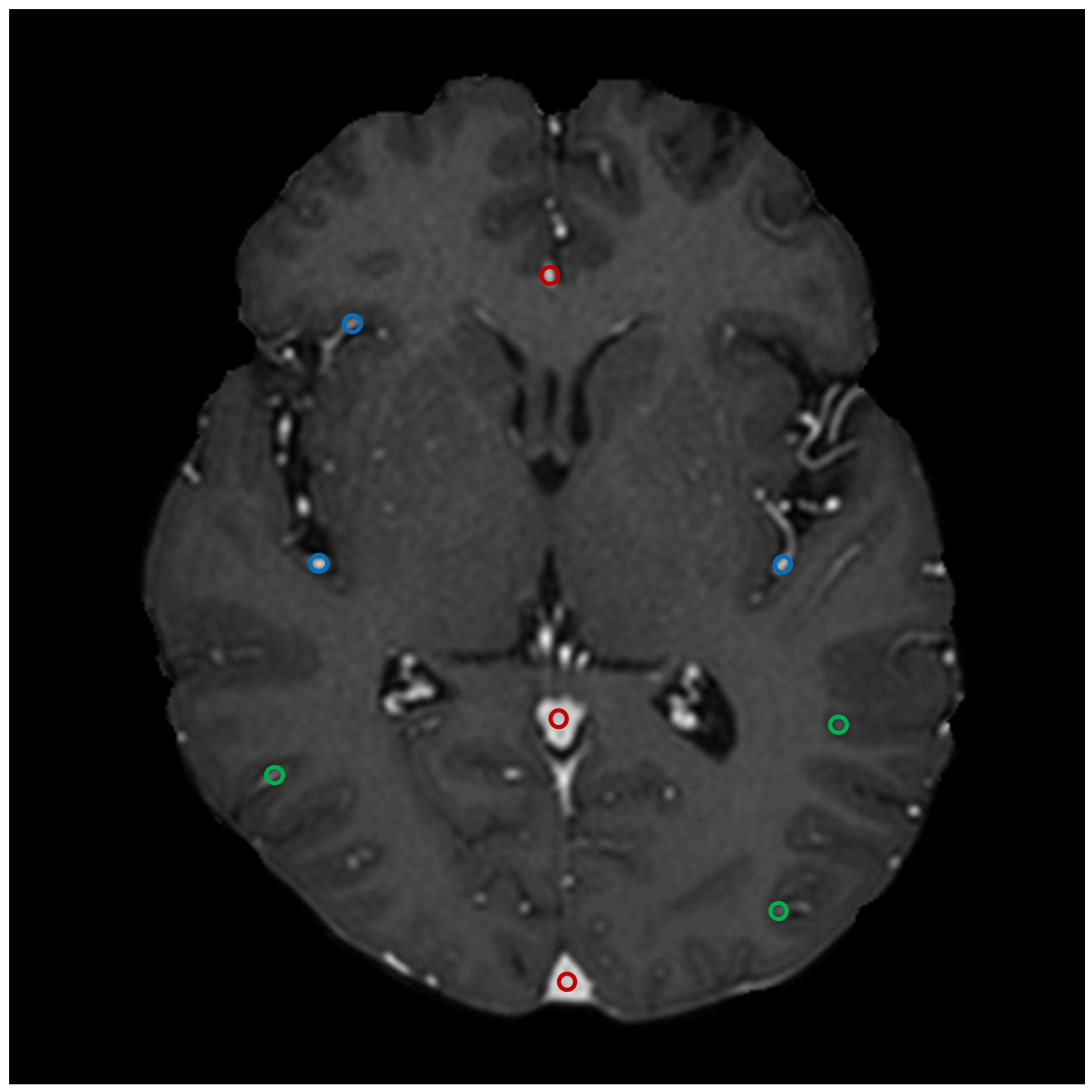
**

**Supplementary Figure S2**. Example of regions of interest (ROIs) for evaluation of blood vessel suppression in a single axial image at the level of the thalamus. Three red ROIs represent type 1 blood vessels (anterior cerebral artery, superior sagittal sinus and vein of Galen); three blue ROIs represent type 2 blood vessels (middle cerebral arteries), and three green ROIs represent type 3 blood vessels (small cortical branches).


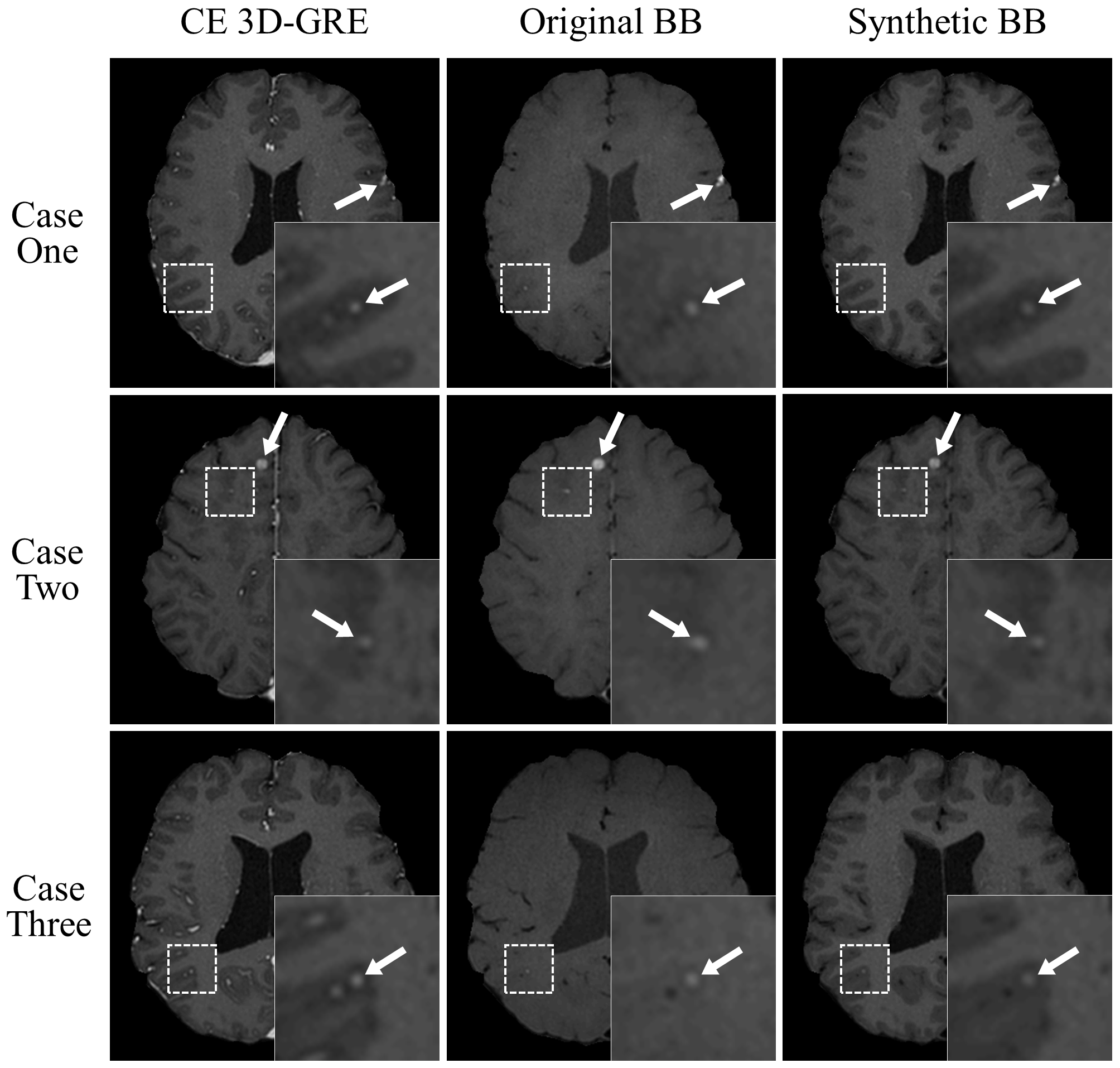


**Supplementary Figure S3**. Results from original and synthetic BB images. Each column represents (from left to right) a CE 3D-GRE image, an original BB image and a synthetic BB image produced by the auto-labelling technique. The rows represent different patients with metastases of different sizes. In all cases, metastases are well observed on both original and synthetic BB images.

**
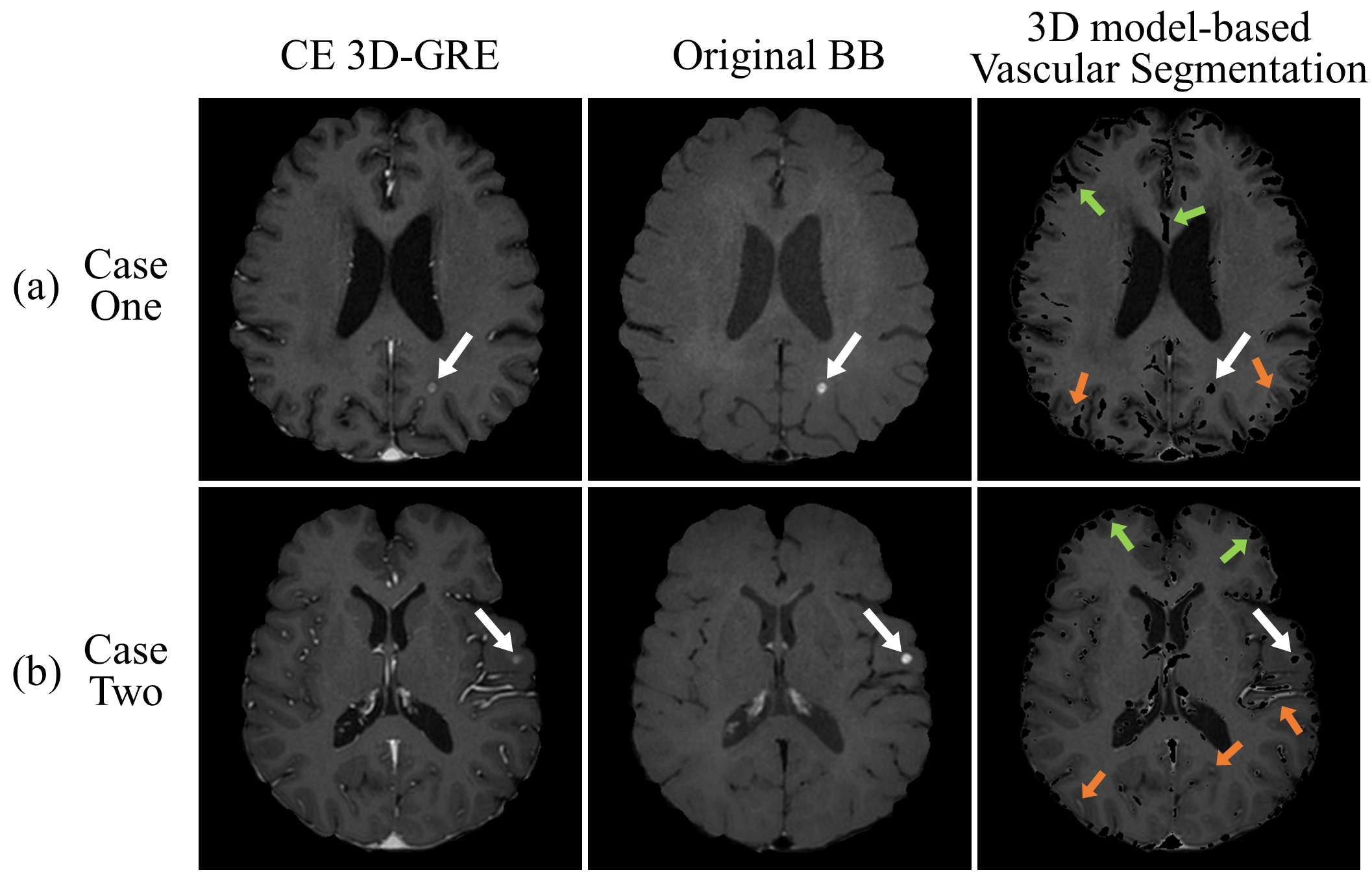
**

**Supplementary Figure S4**. Results of 3D model-based vascular segmentation method on CE 3D-GRE images. In both case one and two, CE 3D-GRE and original BB images demonstrate high signal intense lesions in the left parietal lobe and left temporal lobe, respectively, which were extracted along with blood vessels on 3D model-based vascular segmentation images (white arrows). Although most of blood vessels were extracted using this method, many small cortical branches (type 3) close to the cortex were not extracted and remained (orange arrows). Also, many areas of WM were regarded as vessels and extracted (green arrows).


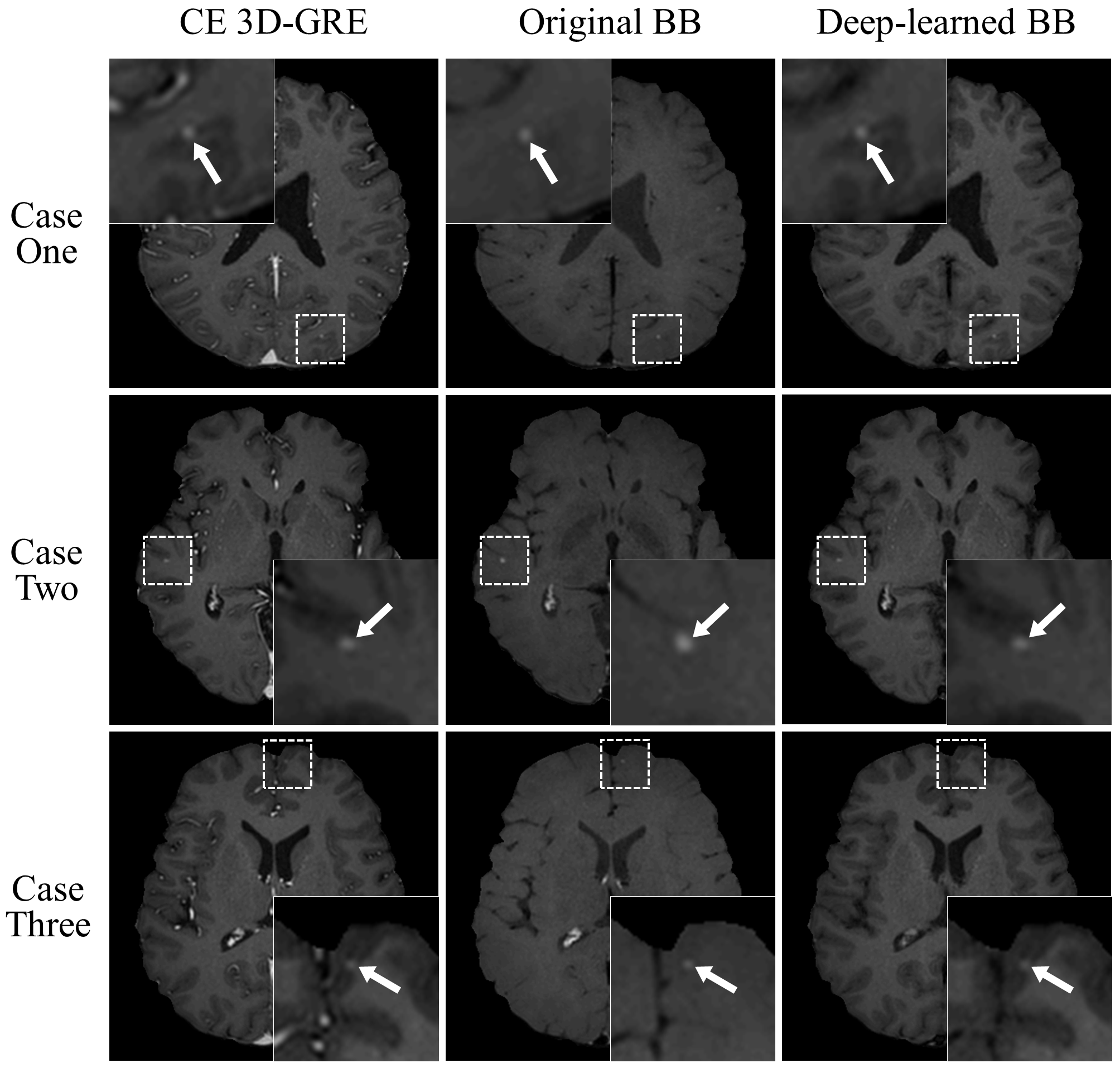


**Supplementary Figure S5**. True positive results for tiny lesions (<2mm) from original and deep-learned BB images. Each column represents (from left to right) a CE 3D-GRE image, an original BB image and a deep-learned BB image produced by the deep-learning process. The rows represent different patients with tiny lesions. These tiny lesions are not easily identified when only CE 3D-GRE images are provided. In all cases, tiny lesions are well observed on both original and deep-learned BB images.


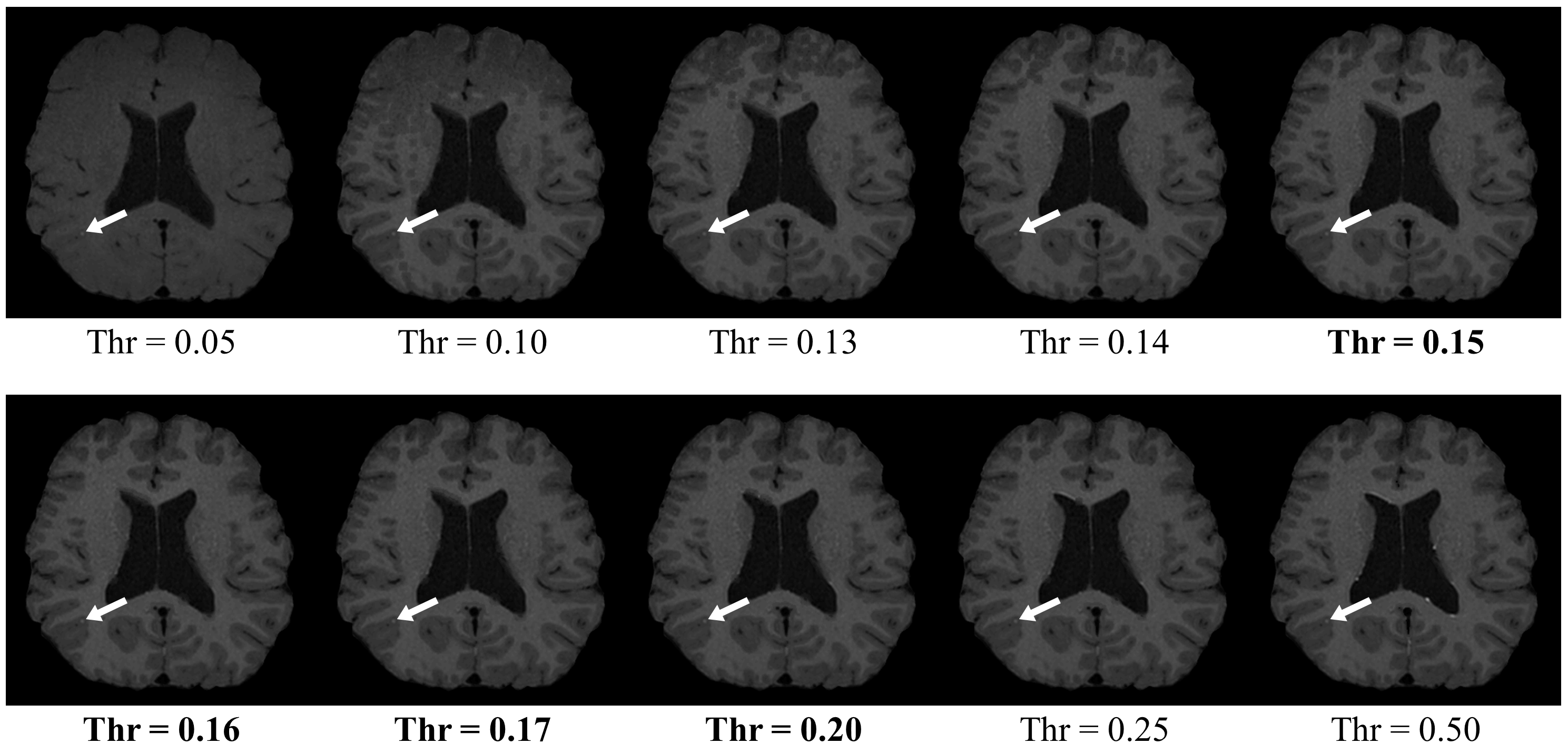


**Supplementary Figure S6**. Examples of synthetic BB images with different threshold values. In all cases, metastases are well observed on synthetic BB images. When threshold value is in range from 0.15 to 0.20, blood vessels are well suppressed while metastases are retained.


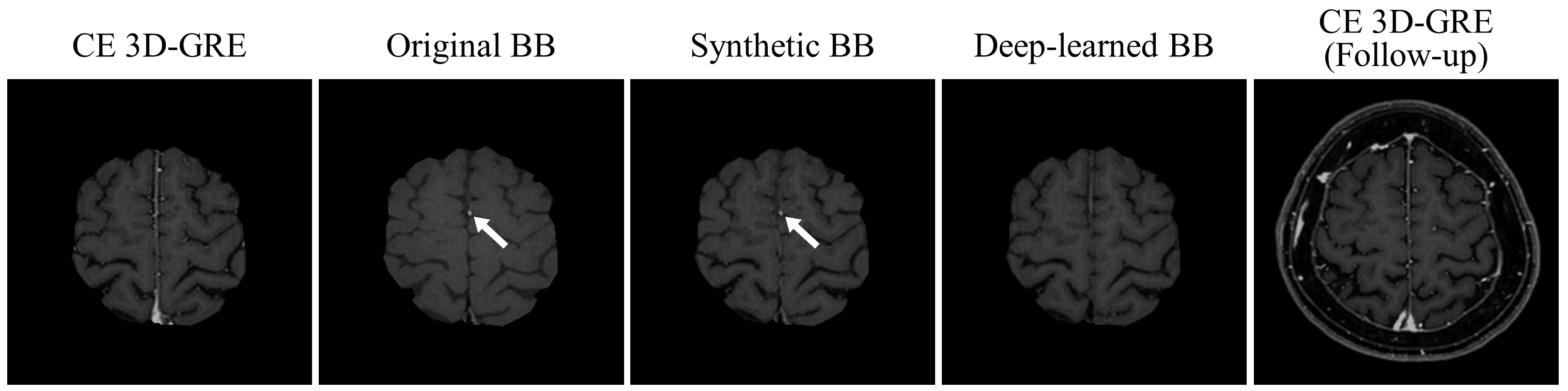


**Supplementary Figure S7**. False positive results from original and synthetic BB images. In this case, the original BB image showed a high signal intensity (arrow) in the left frontal lobe that mimicked a metastasis, but which was due to incomplete vessel suppression in the sulcus, as seen on the contrast-enhanced 3D gradient-echo (CE 3D-GRE) image. The synthetic BB also showed a high signal intensity (incomplete vessel suppression). In contrast, the deep-learned BB image showed no abnormality in the corresponding area. It is confirmed that there is still no metastatic lesion suspected on follow-up scan after 4 months.
